# Notices for July

**Published:** 1869-07

**Authors:** 


### NOTICES FOR JULY.

"The Temperance Speaker," a 16mo of 288 pages, published by The National Temperance Society, and Publication House, 172 William Street, New York, under the industrious and judicious agency of J. N. Stearns, is one of a series of very valuable books, more or less directly in the interest of the great cause of Temperance and Society; and individuals who desire to purchase, for gratuitious distribution, any of these volumes, are counselled to call at the Society's rooms. For the year ending May 1, 1869, over twenty-six million pages of temperance reading were printed, and all good citizens can but be well-wishers of the Society. The above named contains Twenty-three Dialogues, suitable for a variety of occasions; Thirty-five Speeches or Recitations in Prose; and nearly Ninety in Poetry, containing vital Temperance truths and appeals, which should be committed to memory and spoken in every School, Division, Lodge, Temple, or Public Meeting in the Land.

The issues of the American Tract Society, 150 Nassau St., New York, are well worth the attention of all denominations of Christians, as their publications are wisely and scrupulously unsectarian, and are written by men of various sects. "Jesus on the Holy Mount," by Rev. Joseph Sanderson, D.D., pp. 278, is an admirable practical and devotional exposition of that most significant historical incident, and Christian people of all names will be made happier from its perusal.

Lindenwood, or Bertha's Revenge, by Mrs. S. E. Dawes, pp. 446, is one of the "Life Illustrated" series, and well merits the designation, and is well adapted to interest and instruct the young. Also,

The Parables of Our Lord, explained and applied by the Rev. Francis Bourdillon, M.A., Rector of Woolbeding, Sussex, England. We heartily commend the 12mo of 320 pages, large print, to the perusal of all those who love to have the Bible explained to them, whose delight is in the law of the Lord. Above all books purchased for the family, those which truly unfold the meaning of the sacred Scriptures, are the safest, best and most instructive ; this book is among the number. It is a delightful volume, being a practical explanation and application of the parables of the Gospels.

American Year-Book, And National Register, for 1869 ; Astronomical, Historical, Political, Financial, Commercial, Agricultural, Educational, and Religious ; a general review of the United States, including every department of Government National and State, with a brief account of Foreign States, embracing educational, religious, and industrial statistics, facts relating to Public Institutions and Societies ; Miscellaneous Essays, Important Events, Obituaries, etc. Edited by David W. Camp, Vol. 1. Hartford, Connecticut, 834 pp., intended to be followed with a similar annual vol. It is wonderful to note what a vast amount of information is included in this volume ; and information which is permanently useful ; as useful in coming years as now for reference ; it is a book which every cultivated family should possess, and could be placed on every farmer's table with great practical advantage ; the lawyer, the politician, and the author will find this year-book an invaluable compend and a mine of literary wealth.

The Dance of Modern Society, 77 pp., 12mo, price 75 cts., has been issued in beautiful type, by Oakley & Mason, Publishers, 21 Murray Street, New York, written by W. C. Wilkinson, who protests against dancing as a fashionable amusement, as now practiced, and every parent who has a daughter will receive much valuable information from reading this very interesting book ; the article was written for a periodical two years ago, and has been so persistently inquired after from various parts of the country ever since, that the publishers have been induced to bring it out in a more permanent form, and in a style fit for any parlor in the land. Mr. W. says plainly that modern fashionable dancing is unhealthful to the body, and is bestial and degrading in its effects on the mind and morals.

THE CHRISTIAN, 60 cents. A large, live, 8-page monthly religious and family paper, containing incidents, records of providences, sketches, music, poetry, *true* stories, pictures, reading for young, old, saints and sinners. No sectarianism, controversy, politics, puffs, pills, or patent medicines. 60 cts. a year; 10 copies $5. For Sunday schools, 10 copies $4. Send 10 cents for 3 specimens *before you forget it.* Vol. 4 begins Jan. '69. More than 1000 pages new, live tracts, for $1. Address H. L. HASTINGS, SCRIPTURAL TRACT REPOSITORY, 19 Lindall st., Boston, Mass.

HANS BREITMANN "About Town." Being the "second series" of Hans Breitmann's Ballads. *All complete in Two Volumes. Price 75 Cents each.* Hans Breitmann About Town, and other new Ballads. *Being the "Second Series of the Breitmann Ballads."* By Chas. G. Leland, author of "Hans Breitmann's Party." With a portrait of Hans Breitmann. One volume, tinted paper. Price 75 cents. Hans Breitmann's Party. With other Ballads. *Being the "First Series of the Breitmann Ballads." Sixth Edition.* By Charles G. Leland. One volume, tinted paper. Price 75 cents. The above two volumes contain everything that "Hans Breitmann" has written up to the present time, and they are creating a greater sensation in Europe and America than any poems ever before published. Everybody should get them at once and read them. Mrs. Southworth's New Novels. "Fair Play." *Sixth Edition is now ready.* By Mrs. Emma D. E. N. Southworth. Complete in one large duodecimo volume of 700 pages. Price $1.75 in cloth, or $1.50 paper cover. "How He Won Her." *Fourth Edition. A Sequal to "Fair Play."* By Mrs. Emma D. E. N. Southworth. Complete in one large duodecimo volume of over 500 pages. Price $1.75 in cloth, or, $1.50 in paper cover. Ann S. Stephens' New Novel. "The Curse of Gold." *Third Edition.* By Mrs. Ann S. Stephens, author of "Fashion and Famine," etc. Complete in one large duodecimo volume. Price $1.75 in cloth; or

$1.50 in paper cover. Above books are for sale by all booksellers. Copies of any of the above books, will be sent by mail, post-paid, on receipt of price by the Publishers, T. B. Peterson & Brothers, 306 Chestnut St., Philadelphia.

THE SCALPEL."—We are pretty sure that there is not an editor in the United States who will not be gladdened at the resurrection of Dixon's Scalpel, in the same familiar garb as of old, and in the internal filling-up, just as sharp, and scathing, and practical as before; slashing away, right and left, regardless of consequences, to friend or foe, only if truth is allowed to have a fair show. Only yesterday we were sorrowing over the demise of the " Scalpel," being moved thereto by a correspondent down east, and a sharp sensible fellow he is, too, because he appreciates what is practical, useful and true. Hear him under date of May 24, 1869: " The five numbers of the Journal of Health I have received, and I have many a laugh while reading them. I have twelve previous volumes, but have enjoyed these last numbers more than ever. I think you improve. How much I owe to you and Dr. Dixon's Scalpel, ten volumes, no one will ever know but myself. To say that these twenty-four volumes were worth their weight in gold, would not be saying too much; they teach a person how to live right, so as to be happy, could gold buy anything more?" The title page runs thus: " The Scalpel, an entirely original quarterly expositor of the Laws of Health, and abuses of medicine and domestic life." 'Who shall guard the shepherds?' (meaning who shall keep the doctors straight, and teach the parents how to bring up their children physiologically.—Ed. H. J. H.) Edited by Edward H. Dixon, M.D., consulting and operating Surgeon, No. 42 Fifth Avenue, New York; price 50 cents a number. All orders must be addressed to the Editor, P. O. Box 3121." The Scalpel, No. 4, vol. 12, is got up in very attractive style by S. W. Green, Printer and Stereotyper, 16 and 18 Jacob St., New York, successor to that veteran printer, whom we all like, who has retired on a handsome fortune and gone abroad with his eldest son to spend a year or two looking at the old world. May he be returned home in good time, safe, and sound, and happy as the day is long, with the same sunny countenance and honest face he took away with him. But " one word more," about what our correspondent says of the Scalpel ——— and! Hall's Journal of Health. " I should like you to see my little four year old girl, for her continued health, not a sick day, I owe through you and Doctor Dixon, more than to any other."

FUN is the best physic; we like fun; we like a good loud ringing, joyous laugh; we have one every day, about something or other, sometimes a dozen of them. If you were to see Horace Greeley and A. T. Stewart walking arm-in-arm on Broadway, you would be struck with the contrast. Horace looks so perfectly joyous, happy and contented; Mr. Stewart's expression of countenance is the personification of sadness. Both of them have more money than they can ever spend on themselves and families; one is the maker of Presidents, the other the maker of millions of money every year. Most rich men look sad, and people jump at the conclusion that they are miserable; but a greater and more common mistake was never made, for the most intense joys in creation give a sad expression to the face. Who ever thinks of a loud guffaw the first time he hugs his sweetheart; and the first kisses of young love, they are too sweet for a smile; the next time you see two lovers walking together by moonshine in the Central Park, and there are hundreds of such couples every evening there, when they do not imagine that any eyes can be upon them, you will not fail to observe that their faces express sadness personified; and yet we know very well that it is not sadness, but bliss, too full for expression. So that what we call sadness in the countenances of the rich, is not the representation of fear for loss of property or anxiety about investments, it is the index of a mind at ease, from a consciousness of being beyond want, beyond the necessity of toil for a living, a consciousness that without a peradventure, "bread shall be given him, his waters shall be sure," so that we "poor shoats," who have to work for a living, need not, in our enviousness, lay the flattering unction to our souls that the rich are unhappy, for it is not so; they enjoy the ability to take their ease, their fine houses, their elegant equipages and their summer excursions, just as much as you think you would do if you had them; with this advantage, if they did lose every thing, they wouldn't grunt or groan, and fuss over it, half as much as you would. But what were we talking about? we have so many interruptions, at least half of them unprofitable, pecuniarily, and the weather is so ovenly, that the mind wanders; but we were talking about fun, its sanitary value. Well, then, send ten cents to the American News Company, 121 Nassau Street, New York, for a copy of "The Comic News," and have a laugh over it.

Gates Ajar, 248 pp., 12mo, price $1.50. Published by Fields, Osgood & Co., Boston, Mass. This book was written by the grand-daughter of Professor Stuart, of Andover, Mass., considered one of the ablest theologians and biblical expositors of his time; the father of the writer of the book is a Professor in the same theological seminary, and is a man of conceded ability, and is emphatically a scholar; so, on the male ancestral side, the young lady can boast of a hereditary descent of great intellectual ability. The ablest religious papers in the land differ as widely as is possible as to the merits of "The Gates Ajar." Some think it a valuable contribution to the religious literature of the times and much needed; others think it will have a pernicious tendency; we propose in a few words plain and decided to state our "think." Judging from the impression made on our own mind while hearing it read, we are of opinion that it is a mischievous book, unfit to come within the walls of any house where a religious educated family abides; we were shocked by its irreverence and its profanities; its childish efforts to bring into ridicule some of the dearest, and longest, and most universally received tenets of the Christian religions. We do not see how any right minded person can listen, without a shock, to the dialogue between the girl and the good old deacon, and the efforts to place him and his doctrines, the doctrines of the Bible, in an absurd and ridiculous light; making herself the philosopher and he the fool. Whatever makes the Providence of God contemptuous, is an unmitigated wickedness. Her affianced was killed in the Rebellion, and under the guise of its being her brother, she writes the book with a rebelliousness of spirit which taints every page; and as if she would die without comfort, she takes it, in the consideration that when she gets to heaven, she will find her was-to-be husband already there, with a posey in his button-hole, a hat askance, mauve gloves, and a beauty of a little cane, twirling it in his hand; one while they will court over again; another, they will take a walk; at others they will lead a pretty little poodle dog along the streets; now and then stop to see the monkeys perform; and feed the little canary-birds; tired of this, they sing a song, thump the piano, play the fiddle and jump Jim Crow generally; these are not the words exactly, but something of this sort is the idea of the book, the leading idea; and yet such papers as the Advance, of Chicago, and the Congregationalist of Boston spend whole columns in glorifying "The Gates Ajar," while such sterling and long tried, and ably conducted weeklies as the New York

Observer and the Christian Intelligencer, regard it as a bad book and a disgrace to the memory of the girl's grandfather. We earnestly trust she will get a better view of the heavenly world, than through a crack in the door, and that while yet in this world she may be able to see and have the ineffable pleasure of feeling, in the contemplation of God's Providence towards her, that "He doeth all things well," so that when the scenes of earth have passed away, the command may be given "The gates wide open fling," that the angel convoy may convey her to her Father's bosom in the skies, "singing glory."

The Annual Report of the Superintendent of Public Buildings, James M. McGregor, Esq., for 1869, is received; it has been drawn up with great care, and embodies a large amount of useful information, and shows that the Superintendent is fully competent for the duties of his office.

The London Lancet is published monthly, simultaneously with the London issue, by the energy and enterprise of Wm. C. Herald, No. 32 Beekman Street, New York, at $5 a year; postage 24 cts. per annum; supplied to the trade by the American News Co., at 121 Nassau Street, New York. The high character of the Lancet, as "authority," by the medical profession of the United States and Canada, is well understood, and it is well worth the patronage of practising physicians, young and old, as it enables them to keep up with the advanced opinions, improvements, and discoveries of the times, both in surgery and medicine.

Cowan's Patent Copying Paper and Manifold Copying-book, for taking from one to twelve indelible copies at one writing, used by Merchants, Brokers, Telegraph Companies, Reporters, etc., for sale by Cowan & Co., Stationers, at No. 60 Exchange Place, New York. A book of 96 sheets is sold for $1.30, and for convenience, rapidity, and correctness in multiplying correspondence or copies of notes, sales, speeches, etc., it is superior to any thing ever yet devised; one single writing makes a dozen copies which are complete the instant the last word is written; this house also supplies merchants and all banking establishments with blank books of every kind, made to order at the shortest notice in the most durable style and at the lowest prices.

Mr. Charles E. Laumann, late of the house of Phelps, Dodge & Co., will, with his well-known courtesy and promptness, execute, with despatch, any orders left with the firm of Cowan & Co., of which he is a member.

The Illustrated Phrenological Journal for June, contains Portraits and Characters of James Harper; Sir John Young, Governor-General of Canada; Richard G. Pardee; R. A. Murray, the accountant; The Planchette Mystery; Quaker Music; Natives of Alaska, portraits; Great Men—Small Heads; Where are the Housekeepers? Principles in Physiology—Digestion; Should Consumptives Marry? Obedience—its importance; Enduring or Enjoying Life; John Folgate, the Centenarian; Face Fancies; What can I do best? The Woman Question; Music; Answers to Correspondents, etc. End of Volume 49; New Volume begins with the next number. Only 30 cents, or $3 a year. Address S. R. Wells, 389 Broadway, New York.

Philosophy of Eating. Albert J. Bellows, M. D., late Professor of Chemistry, Physiology and Hygiene; 344 pp. 12mo. Published by Hurd and Houghton. Cambridge, Riverside press. Fifth Edition. Dr. Bellows has done a public good by the issue of this book which contains a large amount of practical information upon the every day business of eating. The book is sent post paid for two dollars in a post office check addressed to Hurd & Houghton, 459 Broome street, New York, and merits a wide circulation. We will send it free to any one who will send us three subscriptions.

Free Bathing Places for All. While Dr. David P. Holton, 124 West 54th st. is giving his personal services gratuitously, for procuring free baths of fresh or salt water, cold or warm, Dr. Harris, sanitary superintendent of the Metropolitan Board of Health has generously pledged to the cause one thousand dollars. Who of the Stuarts and Stewarts, and Coopers, and Dodges, and Drews, and Lenoxes, and Greenes of liberal New York follow the noble example? Dr. Holton will deliver single or courses of Lectures on Physiology and Hygiene for the benefit of "The Institute of Reward for Orphans of Patriots." Dr. Horace Webster and Marshall O. Roberts with others of our prominent citizens here publicly commended Dr. Holton's Lectures as well worthy of patronage.

